# Role of GRPR in Acupuncture Intervention in the “Itch-scratch Vicious Cycle” Spinal Circuit of Chronic Pruritus

**DOI:** 10.1186/s13020-022-00706-4

**Published:** 2023-01-03

**Authors:** Jia-jia Liu, Xuemei Li, Jing Guo, Shuguang Yu, Sha Yang

**Affiliations:** 1 Acupuncture and Tuina School, Chengdu University of Traditional Chinese Medicine, Chengdu, Sichuan China; 2 Acupuncture and Brain Science Research Center, Chengdu University of Traditional Chinese Medicine, Chengdu, Sichuan China; 3 Key Laboratory of Sichuan Province for Acupuncture and Chronobiology, Chengdu, Sichuan China

**Keywords:** GRPR, Acupuncture, Chronic pruritus, Itch-scratch, Spinal circuit, Ventral tegmental area (VTA), Periaqueductal gray matter (PAG)

## Abstract

Many previous studies have shown the potential antipruritic effect of acupuncture. This paper reviews the antipruritic mechanisms of acupuncture according to these aspects: sample characteristics, detail of intervention, and effects evaluation. The majority of research on acupuncture’s antipruritic effect has focused on primary afferents of the peripheral mechanism. Relatively few studies, however, have addressed the central mechanisms. Combination the latest research achievements of chronic itch, gastrin-releasing peptide receptor (GRPR) in the dorsal horn of the spinal cord may represent the first molecule identified that is dedicated to mediating the itch response and may provide an important therapeutic target for the treatment of chronic pruritic conditions. Therefore, GRPR may be a new target for acupuncture to relieve itch in the future and provide new ideas for acupuncture intervention in the mechanisms of the spinal level of the “itch-scratch vicious cycle” of chronic itch.

**Supplementary Information:**

The online version contains supplementary material available at 10.1186/s13020-022-00706-4.

## Introduction

Itch is an unpleasant sensation inducing the irresistible impulse to scratch. Generally, pruritus is classified as acute or chronic, with chronic pruritus defined as an itch lasting more than six weeks. A fifth of the general population suffers from chronic pruritus at least once during their lifetime, and some populations with skin diseases experience it up to 100% [1]. In chronic cases, itchy skin with burning and stinging sensations [2] impairs patients' quality of life [3] and reduces their productivity [4], causing sleep disturbances [5]. Typically, prolonged intense itch results from an addictive “itch-scratch vicious cycle” [6, 7], potentiating damage to the skin and more itching.

Chronic pruritus can be caused by many conditions, including skin diseases, immune system diseases, nervous system disorders, or internal organ metabolic disorders [8]. Histamine-dependent itch in a few instances, antihistamine drug therapy is ineffective for the majority of chronic itch. There has been considerable advancement in the treatment of chronic pruritus through the development of biologics and κ-opioid receptor agonists (KORs). However, biologics are expensive and KOR agonists are fraught with risk [9]. It is possible to distinguish mechanical itch from chemical itch based on peripheral input, the latter being further divided into histaminergic and non-histaminergic. Gastrin-releasing peptide receptor (GRPR) as the G protein-coupled receptor (GPCR), is the first itch-specific receptor in the spinal cord of mice [10]. GRPR-expressing neurons are the key parts of itch information from the periphery to the brain [11], playing an important role in the spinal cord loop of chronic pruritus [12, 13], with other neuro mediators such as 5-hydroxytryptamine (5-HT) involving “itch-scratch vicious cycle” in the process of chronic pruritus on the neural loop of spinal level [14]. GRPR as the core of itch transmission is an attractive target for antipruritic intervention [15]. The effect of acupuncture inhibits the GRPR expression preliminary confirmation received [41]. The purpose of this paper is to discuss the possible mechanism of acupuncture intervention at the spinal level of the “itch-scratch vicissitudes” from the perspective of GRPR regulation and release.

## The mechanism of acupuncture treatment for chronic itch

Acupuncture has a long history of antipruritic effects as a traditional complementary and adjunctive therapy, with the related study first reported in 1984 [16]. Many types of researches have shown that acupuncture relieves pruritus while improving multidimensional quality of life issues such as mood, work, and life after the disease [17–19]. The mechanisms of acupuncture to cure chronic pruritus are unknown [20–22]. In atopic dermatitis patients, acupuncture decreased itch-evoked activation in the insula, putamen, premotor and prefrontal cortices [23]. After the integrated intervention (Including acupuncture), chronic spontaneous urticaria (CSU) patients exhibited reduced the left putamen, caudate, accumbens, and thalamus with resting state functional connectivity (rs-FC) [24]. Acupuncture enhanced positive functional connectivity of the putamen-the posterior part of the midcingulate cortex (pMCC) in against histamine-induced itch of healthy volunteers [25]. According to the literature, the mechanism of acupuncture for itch in the central mechanisms has been comparatively little studied, mainly about some brain regions in the patient [23]and microglia in the mouse spinal cord [26], therefore the mechanism of acupuncture used to treat simple itching above the spinal cord need more evidence. With the deepening of the studies on the animal mechanism of chronic pruritus “itch-scratch vicious cycle” in the spinal cord and central circuit, taking advantage of acupuncture’s ability to treat chronic pruritus “itch-scratch vicious cycle” makes it all possible.

**Fig. 1:**
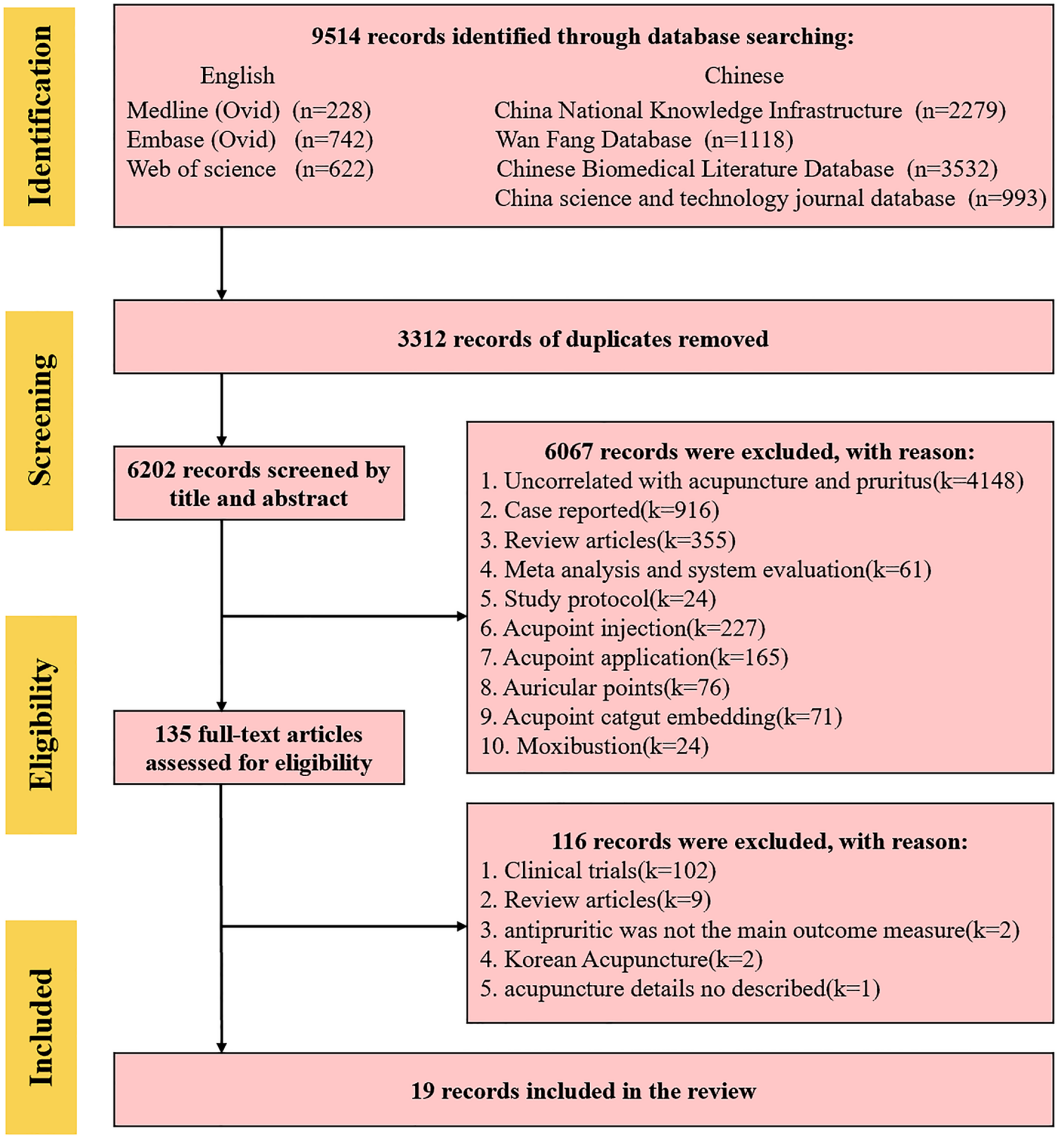
Flow chart of search and selection process of included studies

Seven online databases were searched by two authors independently from inception to October 2022: PubMed, EMBASE, Web of Science, China National Knowledge Infrastructure (CNKI), Wan Fang Database, China Science and Technology Journal Database (VIP), Chinese Biomedical Literature Database (CBM). The literature searches were constructed around medical search headings (MeSH) for acupuncture and pruritus, and only animal studies were included. According to the needs of each database, the search strategies were made appropriate adjustments. The specific search strategies were listed in Additional file 1. A total of 9514 relevant studies were retrieved. A total of 3312 duplicated studies were removed, and 6202 studies were removed after screening titles and abstracts. One hundred and sixteen studies were excluded after reading the full text. Finally, 19 animal studies (27–45) were included. The specific study screening process and results are shown in Fig. 1. At present, most studies on the mechanism of acupuncture intervention on pruritus of animals used Sprague–Dawley (SD) rats or BALB/c mice as the itching model. The specific modeling methods and intervention details (points used, needle type, depth of insertion, needle insertion manipulation, needle stimulation (manual or electrical), needle retention time, and several treatment times) are shown in Table 1.

**Table 1:** Summary information of itch modeling and needle details for acupuncture intervention

References	Animal Type	Disease vector	Modeling method	Point selection	Needle	Needle depth		Needle Angle	Acupuncture technique /stimulation Parameters	Frequency	Needle retention time	Course of treatment
[27]	Rat	Rat model of neurodermatitis	Combining chronic mild unpredictability stress with local skin friction stimulation	DU20, bilateral GB20	0.35 × 25 mm	0.3 inches		\	\	\	\	Continuous acupuncture treatment for 14d
[28]	Mouse	Histamine-dependent pruritus mouse model	Injected with histamine phosphate subcutaneously into the neck and back	Bilateral SP10, LI11 and LI4	No.30needle	5 mm		Puncture straightly	Lifting, thrusting, and twirling	\	20 min	once daily for 2d
[29]	Mouse	Histamine and chloroquine-induced itch model in mice	Subcutaneous injection of histamine phosphate or chloroquine at the shaving area of the nape	Bilateral SP10, LI11 and LI4	\	2.5 ~ 3 mm		Puncture straightly	Lifting, thrusting, and twirling	\	20 min	Once daily for 2d
[30]	Mouse	Chloroquine-induced itch model in mice	Subcutaneous injection of chloroquine into the shaving area back of the neck	Bilateral SP10, LI11 and LI4	\	5 mm		Puncture straightly	Lifting, thrusting, and twirling	\	20 min	Once daily for 3d
[31]	Mouse	Atopic dermatitis mouse model	Apply 100 μl of 2% DNCB acetone solution to the shaved abdomen and ears of mice	Bilateral PC6, LI11	\	1 ~ 2 mm		\	Intensity 1 ~ 2 mA (rat limbs shaking slightly), sparse and dense waves, frequency 2/100 Hz, alternating	2/100 Hz	20 min	Once alternate days for 9 times
[32]	Mouse	Mouse model of allergic contact dermatitis	Apply 200 μl of 1 ~ 2% DNCB acetone solution to the shaved abdomen, extremities, and ears of mice	PC6, ST36	Disposable acupuncture needles	4 mm		Puncture obliquely	Sparse and dense wave, 5–10 times/s, frequency 2/100 Hz, current intensity 1 ~ 2 mA(the mouse limb muscle slight tremor)	2/100 Hz	30 min	Once alternate days for 3 weeks
[33]	Mouse	Compound 48 /80 induced itch model in mice	Compound 48 /80 injected subcutaneously into the rostral part of the back	SP10, LI11	\	\		\	Current intensity 2 mA, frequency 2/15 Hz	2/15 Hz	30 min	1d
[34]	Rat	Model rat of type I hypersensitivity	The mixture of 1 mg OVA and 1 ml Al(OH)3 suspension injected into the abdominal cavity of the rat	Bilateral SJ10, SJ11, non-channel-non-acupoint	\	\		Penetrative needling	Sparse and dense wave(2 Hz/30 Hz), current intensity 2 mA, output voltage 2 ~ 4 V(local light tremor), with Zhiyin bloodletting daily	2/30 Hz	30 min	14d
[35]	Rat	Model rat of type I hypersensitivity	The mixture of 1 mg OVA and 1 ml Al (OH)3 suspension injected into the abdominal cavity of th e rat	SJ10. (other points not described)	\	\		\	Electroacupuncture needled	\	\	14d
[36]	Mouse	Compound 48 /80 induced itch model in mice	Compound 48/80 injected subcutaneously into the center of the neck	SP10, LI11	\	\		\	Current intensity 3 mA, frequency 2/15 Hz	2/15 Hz	30 min	1d
[37]	Rat	5-HT-induced itch model in rat	Intradermal injection of 10 ml NS or 2% 5-HT at the nape of the neck	LI11, LI4, ST36, SP6	\	Quchi 4 mm, Hegu 1 mm		Puncture straightly	Post-anesthetization electroacupuncture (sparse and dense waves of 4–16 Hz), and the intensity is gradually increased from 1 V pulse voltage to 3 V	4–16 Hz	1 V 10 min 2 V 10 min 3 V 20 min	Once
[38]	Rat	Model rat of type I hypersensitivity	Primary immunization and excitation of rats with an intraperitoneal injection of ovalbumin the topical	EASJ10, SJ11, BL67 bloodletting	\	\		\	Sparse and dense waves 2 Hz/30 Hz, Current intensity 2 mA, output voltage 2 ~ 4 V(local light tremor)	2/30 Hz	20 min	Once daily for 14d
[39]	Mouse	MC903(a vitamin D analog) induced atopic dermatitis model	Application of MC903 to the mouse cheek	LI11	0.18 × 8 mm; Haenglim-Seoweon Acuneedle Co., Korea	2-3 mm		\	The needle was twisted for 30 s at a rate of twice per second and immediately removed	\	30 s	Preventive acupuncture-treated group 11d therapeutic acupuncture-treated group 4d
[40]	Mouse	Acetone and diethyl-induced chronic dry skin itch mice model	A piece of gauze infiltrated with a volume ratio (1:1) of acetone and diethyl ether was applied to the shaved portion of the mouse neck for 15 s and then another piece of gauze infiltrated by distilled water was used for another 30 s, twice a day for 9 consecutive days	Left side: LI4, LI11, SP10,ST36	\	2-3 mm		\	The current was delivered through Han’s acupoint nerve stimulator (LH202, China), and frequency was 100 Hz, the intensity of 1 mA or 3 mA, and the wave width was 0.3 ms	100 Hz	30 min	EA was performed once every other day, A total of five treatments of EA were performed
[41]	Mouse	The mixture of acetone and diethyl ether (1:1) induced the dry skin mice model of chronic itch	A mixture of acetone and diethyl ether (1:1) was applied to the shaved area by a piece of wet gauze at the neck for 15 s and then using another gauze infiltrated by distilled water for another 30 s, twice a day for 9 days consecutively	Left side: LI4, LI11	0.25 mm outer diameter	2-3 mm		\	EA (1 mA) was administered with different frequencies (2, 15, or 100 Hz), a modified current constant Han’s Acupoint Nerve Stimulator (LH202; Huawei Co., Ltd., Beijing, China), and the shaking of the left forelimb muscle as the criterion for the success of the EA	2/15/or 100 Hz	30 min	EA treatment once every other day for 5 times
[42]	Mouse	Morphine-induced pruritus model	intrathecal morphine injection to induce pruritus	Bilateral LI11,SP10	7 × 0.20 mm;Huatuo,Suzhou,China	5 mm		Vertically	An intensity of 2 mA and a frequency of 2/15 Hz dilatational wave	2/15 Hz	30 min	EA preconditioning was conducted each day for 5 consecutive days before modeling
[43]	Mouse	DCA and GTI induced scratch model	Mice were injected subcutaneously with GNTI (0.3 mg/kg) or DCA (8.3 mg/kg) to the back of the neck	ST36, LI4, LI11	\	2 mm		\	EA Trio 300 stimulator (Ito, Japan) at an intensity of 2 mA for 20 min at 2 Hz, using a 150 μs pulse width	2 Hz	20 min	\
[44]	Rat	Capsaicin-induced model of atopic dermatitis	Capsaicin (50 mg/kg) was subcutaneously injected into rat pups 48 h after birth	Six acupoints (bilateral BL13, and unilateral LI11,ST36,SP10,SP6)	Disposable intradermal needles (0.12 × 4 mm, stainless steel, Haeng Lim Seowon Medical)		\	Inserted oblique	A frequency of 120 Hz, mixed (120 + 12 Hz), intermittent (10 s stimulation and 5 s no stimulation) EA intensity was controlled 75 ~ 100Vp-p (9.5 mA) such that rats were comfortable and did not squeak, but weak muscle twitches were induced	120 Hz, mixed (120 + 12 Hz)	30 min	\
[45]	Rat	Serotonin-Evoked Itch	An intradermal of 2% serotonin (20/μl) injected into the rostral back	ST36, SP6,LI4, LI11 and the itchy points (pruritic region);	\	5 mm		\	EA:2 Hz or 120 Hz. Voltage: 3 V-6 V Rats were comfortable and do not squeak but had weak muscle twitches	2/120 Hz	30 min	\

### Modeling methods

The role of acupuncture in the mechanisms of pruritus has mainly been studied in animal models of itching caused by pruritogens, or exploring acupuncture based on the current disease for relieving the degree of itching. Histamine, chloroquine, 5-HT, compound 48/80, ovalbumin (OVA), aluminum hydroxide (Al (OH)_3_)) and other drugs were mainly injected of drug-induced itching model in acupuncture research, histamine phosphate, chloroquine, and compounds 48/80 in used top 3. Histamine phosphate and compound 48/80 are used for constructing an animal model of histamine-dependent itch [46]. The chemical mediator histamine is present in both basophils and mast cells [47, 48]. Cells release histamine after degranulation to provoke itch when the body is stimulated by immunologic or nonimmunologic factors. Compound 48/80, a polymer of p-Methoxy-N-methylphenethylamine monomers, induce the release of several inflammatory factors and chemical mediator to elicit skin itch [49], in addition, to mimic more stably stable animal models of skin itch. Chloroquine as an antimalarial drug accompanied by cutaneous adverse reactions [50], was used to construct histamine-independent itch models. Based on a particular disease such as neurodermatitis, the animal model used the stress method of chronic mild unpredictability with local skin friction stimulation to study the mechanism of acupuncture for pruritus. Histamine, compound 48/80, and chloroquine are commonly used as pruritogenic agents with the intervention course in 1–3 days usually, other pruritogenic agents provoke itching model with the intervention course in 7–14 days. The allergic dermatitis model elicited by DNCB acetone solution [32] and the neurodermatitis model elicited by chronic mild unpredictable stress method and local skin friction [27] had long intervention courses in the 14–21 days. Compared to simple itching and scratching models, the acupuncture treatment course is shorter, and it is associated with itch-scratch discussed in a specific disease [51].

### Acupuncture details

Acupuncture points used for the itch model shown in Table 1, include Quchi (LI11), Xuehai (SP10), Hegu (LI4), Zusanli (ST36), Tianjing (SJ10), Sanyinjiao (SP6), Neiguan (PC6), Qinglingyuan (SJ11), Baihui (GV20), Fengchi (GB20), Weizhong (BL40), Geshu (BL-17), Feishu (BL13), Zhiyin (BL67), localized itch. LI11 (14/19), SP10 (8/19), and LI4 (8/19) rank in the top 3 of acupoint selection. The electroacupuncture of sparse and dense waves as the main intervention make local tremor lightly, with a sparse wave of 2 Hz, a dense wave of 30 Hz/100 Hz/120 Hz, or sparse and dense waves of 416 Hz in 5–10/s, a current of 1–2 mA, the output voltage of 1–4 V. The single intervention time is usually 20 or 30 min, according to different design purposes, and the duration of treatment varies from 1 day to 3 weeks. On the time point of electroacupuncture intervention, several studies have constructed an itching model after electroacupuncture pretreatment to examine the potential mechanism of the treatment [39, 42]. The timepoint, duration, and course of non-electroacupuncture intervention are adjusted depending on the observation purposes and the differences of modeling methods, such as the observation of the antipruritic of immediate effect [28–30, 33, 36, 37].

### Effect of acupuncture on itch symptoms

Acupuncture treatment of pruritus mainly focused on general state observation (emotional reactions, mental states, diet and sleep of status, fecal traits, and skin lesions in the local modeling area) and behavioral examination (most experiments preferred scratching symptoms). Some experiments focused on depressive behavior assay (open field test, sugar-water consumption test, food consumption measurement, and body mass change measurement, the tail-flicking time), skin wheal and optical density. The itch and scratching symptoms haven't been recorded by uniform standards, but most studies are conducted in quiet environments without humans for 30–60 min. The pruritic behavior was judged by the number of scratches: a complete scratching action was considered when the mice left the bottom of the box with their hind paws and scratched the skin until they dropped their hind paws or licked their hind paws, regardless of number of scratches during this period [52].

Based on Table 2, detection markers in studies of acupuncture treated pruritus mainly used peripheral mediators: amines, endogenous peptides, and prostaglandins. The nerve conduction of pruritic mediators plays an important role in pruritogenesis, mediators conducting pruritus by stimulating class C nerve fibers or via combination with receptors on cutaneous sensory nerve fibers [53], also having an important role in the “itch-scratch cycle” [54]. Positive expression of substance P in the epidermis and dermis of the skin, the sensory nerve fiber endings containing substance P have a bidirectional conduction function. One by activating Neurokinin-1(NK1) receptors on the unmyelinated C fibers conduct itch directly [55]; another by promoting the release of other pruritic mediators such as histamine, leukotrienes, β-endorphins, and inflammatory cytokines Interleukin(IL)-2 and IL-4 conduct itch indirectly [56]. Peripheral mediators such as histamine, prostaglandins, and inflammatory mediators can be involved in pruritic signal formation by activating Transient receptor potential vanilloid 1(TRPV1) expressed by c-nerve fibers and keratin-forming cells and mast cells, which generate and amplify pruritic signals and decrease the itch threshold [57]. The cytokines IL-2 and IL-4 also enhance pruritic neuronal excitability broadly to involve in pruritic pathway transmission [58]. Regarding sample collection except for serum and skin lesions, the spinal cord and medulla oblongata has been gradually considered, laying the experimental foundation for future studies to explore the mechanisms of acupuncture for pruritus at higher-hierarchical centers.

**Table 2:** Commonly used detection methods and indicators in animal mechanism research of acupuncture treatment of pruritus

Detection method	Detection indicators	Effects evaluation indicators
Enzyme-linked immunosorbent assay (ELISA)	Substance P(SP) [27, 31], 5-HT [28], 5-HT receptor [28], IL-4 [32], Interferon-gamma (IFN-γ) [32], histamine [34], Prostaglandin E_2_(PGE_2_) [34], ionized calcium-binding adapter molecule 1(Iba1) [43] TNF-α [43], IgE [44], IL-1 [42], IL-6 [42], IL-12 [42], IL-10 [42], Tumor necrosis factor-α (TNF-α) [42, 43], Serum corticosterone [39]	Scratch behavioral tests [27, 28, 31, 34], general state, depressive-like behaviors test (open field test, sugar water consumption test, food consumption measurement, and body mass change measurement) [27], lesion, pathomorphological, thickness and dermatitis of skin [27, 31], weight measurement [31]
Immunofluorescence	Beta-endorphinergic (β-EP) neurons [29], 5-HT neurons [30], cannabinoid receptors type 1 (CB1) receptors [40], 5-HT neurons [40], GRPR neurons [40], Dynorphin (DYN)-A [41]	Scratch behavioral tests [30, 40, 41]
Immunohistochemistry (IHC)	IL-4 [38], IFN-γ [38], Scar tissues [44]	Behavioral tests (including scratch) [38], skin thickness, and dermatitis scores were obtained [44]
Western-blot(WB)	Beta-endorphinergic [29], 5-HT 2B receptor protein [29], DYN [31, 44], Cyclic AMP response-element binding protein (CREB) [39], phosphor-CREB (pCREB) [39], deltaFosB(ΔFosB) [39], brain-derived neurotrophic factor (BDNF) [39], tyrosine hydroxylase(TH) [39], dopamine D1A receptor (D1AR) [39], Dopamine and cAMP-regulated phosphoprotein (32 kilodaltons) (DARPP-32) [39], pDARPP-32 [39], Beta-actin [39],CB1 [40],CB2 [40],(glyceraldehyde-3-phosphate dehydrogenase) GADPH [40], GRPR protein [41],DYN-A Protein [41]	Scratch behavioral tests [29, 39], anxiety- and depressive-like behaviors [39]: elevated plus-maze (EPM), open-field tests (OFT), and tail-suspension test (TST). Atopic dermatitis (AD)-like skin lesions with epidermal thickening [39]
Pathological examination	Lesional tissue [32]	Scratch behavior tests [32], skin lesion [32], weight measurement [32],
Colorimetry	Degranulation of mast cells [44]	Scratch behavior tests [44], skin thickness, and dermatitis scores were obtained [44]
Confocal image analysis	Iba1 localization [43]	Scratch behavior tests [43]

In summary, studies on the mechanisms of acupuncture regulating the itch pathway focus on the periphery currently, and studies on the mechanisms of higher-hierarchical centers are gradually deepening, but related contents are not sufficient. Correlation research had suggested that electroacupuncture may inhibit itch signaling in the center by promoting the release of prednisolone (DYN) in the spinal cord, activating massive κ-receptors, which on the one hand diminished itch signaling in the center, on the other hand alleviating the stimulation on peripheral itchy nerves by suppressing the release of endogenous peptide receptor SP [31]. The latest studies were shown electroacupuncture through activating KOR in the dorsal horn of the spinal cord inhibiting GRPR expression to alleviate chronic pruritus [41]. Therefore, whether the modulatory effect of acupuncture on GRPR can inhibit the signaling in the “itch-scratch vicious cycle” to treat chronic pruritus will be a possible mechanism for the future treatment of chronic pruritus by complementary alternative medicine. In this paper, we will explore the possible mechanism of GRPR in acupuncture intervention in the “itch-scratch vicious cycle” from the spinal cord and above neural pathways of chronic pruritus, to provide new ideas for future acupuncture treatment of chronic pruritus “itch-scratch vicious cycle”.

## Role of GRPR in itch spinal cord loops

The 2007 study revealed that GRPR may be the first molecule dedicated to the regulation of pruritus sensation in the dorsal horn of the spinal cord [10], and subsequent studies found that, unlike the traditional spinal thalamic tract, GRPR nerve conduction pathways constitute a long-term marker line of pruritus sensation in the spinal cord [59]. GRPRs are activated by a variety of G Protein-Coupled Receptors (GPCRs) that conduct pruritic signals to induce scratching. Morphine is commonly used for pain relief but can induce pruritus, and studies have shown that morphine provokes itch by triggering the internalization of GRPR and mu-opioid receptor (MOR)-1D [60, 61]. Pruritus-specific output is increased by Gastrin-releasing peptide (GRP)-GRPR signaling mediated by 5-HT1A receptors [62]. Atopic dermatitis is caused by the activation of the GRP/GRPR pathway, which is mediated by IL-22 [63]. Although various neuropeptides such as substance P and B-type natriuretic peptide (BNP) are related to GRPR in spinal pruritic signal propagation, the related pathways and their roles need to investigate further. However, recent studies suggested that both mechanical and chemical pruritus brought about by light touch can converge on spinal GRPR neurons transmitting to the center [64], so GRPR may be the integration point of various pruritic spinal pathways.

## The “itch-scratch cycle” activates brain regions and spinal cord loops

Scratching is a vital factor in maintaining chronic pruritic symptoms and decreases the quality of life of patients [65]. Patients with chronic pruritus experience severe itching and a strong desire to scratch, and a vicious “itch-scratch cycle” can easily form as the affected individual struggles to resist the strong impulses of itching and scratching. This scratching often leads to tissue damage to constitute conscious self-harm [66], but in the chronic itching situation, the pleasure from scratching to relieve the itch is hedonic and activates the central reward system significantly. There are unmyelinated C nerve fibers in the skin that are responsible for itching. These fibers form synapses and are connected to the thalamus and parabrachial nucleus in the brain. After the pruritic signal reaches the thalamus and parabrachial nucleus, several areas in the brain are activated, including the prefrontal cortex (PFC), supplementary motor area (SMA), premotor cortex (PM), primary motor cortex, primary (SI) and secondary somatosensory cortex (SII), parietal cortex, cingulate cortex [67], precuneus, insula [66–69], basal ganglia, midbrain and cerebellum [70]. As shown in Table 3, brain regions closely associated with itch-scratch are closely linked.

**Table 3:** Brain regions closely related to itching and scratching circulation

References	Brain regions
[39]	Nucleus accumbens, dorsolateral striatum, ventral tegmental area
[40]	Ventrolateral periaqueductal gray (vlPAG)
[71]	Anterior cingulate cortex-dorsomedial striatum (ACC-DMS)
[72]	Supplementary motor area (SMA), premotor cortex (PM), the primary motor cortex (PMC), middle cingulate cortex (MC), caudate nucleus (CN)
[67]	Insula, cingulate cortices, basal ganglia, frontoparietal control network (superior parietal lobule and dorsolateral prefrontal cortex)
[68]	Anterior cingulate, insula, claustrum, hippocampus, nucleus accumbens
[70]	Dorsolateral prefrontal cortex (dlPFC), cerebellum
[73]	ACC、posterior cingulate cortex(PCC), retro splenial cingulate cortex, dlPFC, caudate nucleus, putamen, PMC, primary somatosensory cortex, superior parietal lobe
[6]	Ventral tegmental area (VTA)
[74]	ACC, insula, VTA, periaqueductal gray matter (PAG), basal ganglia

**Fig. 2:**
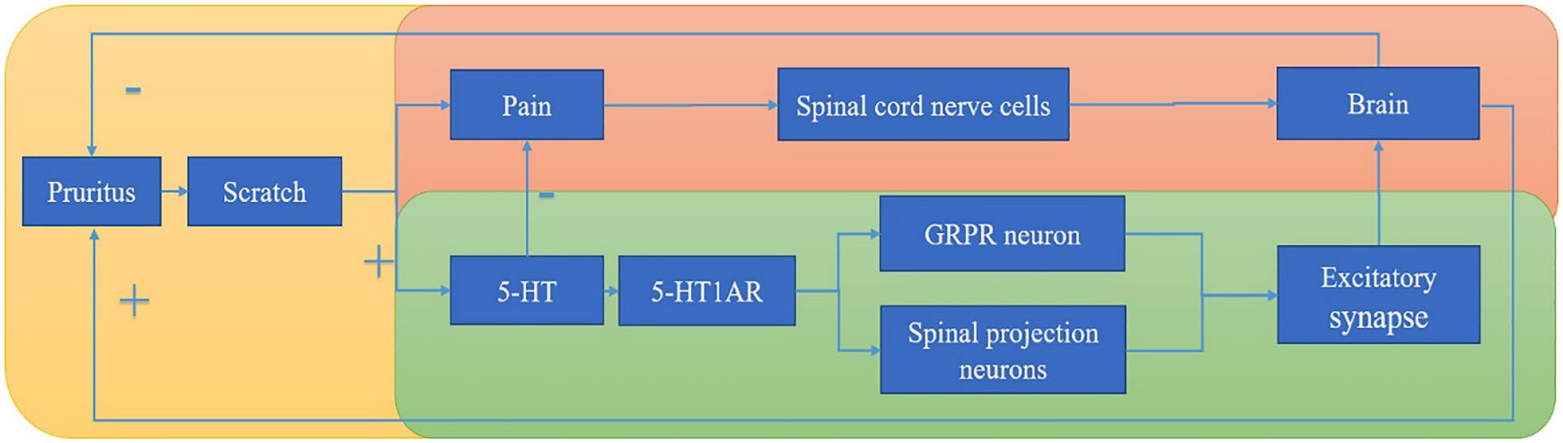
Itch provoking scratching caused pain and pruritus will be together transmitted to the center. Pain will be conveyed to the brain via spinal nerve cells, feedback to the periphery to reduce itching. Itch through 5-HT receptor activation GRPR + neurons and spinal projection neurons aggregated to excitatory synapses transferred to the brain, and the brain feedback to the periphery to aggravate itching causing a new “itch-scratch cycle”

Itch perception is regulated by multiple brain regions in central processing and further via efferent nerves trigger scratching as shown in Table 3. Three nodal findings emerged from studies on the chronic pruritic “itch-scratch cycle”: First, at the spinal cord level, the “itch-scratch vicious circle” principle indicates that when itching is felt, the pain from scratching the skin is transmitted to the brain through nerve cells in the spinal cord, interfering with the itch signal and temporarily suppressing the itching sensation. It's the pain that hides the itch. In this case, pain is activated with itch suppressed, since pain and itch are not identical, but they share a neural circuit in the same area of the brain. As a result of scratching, itch sensitivity is reduced, thus eliminating itch for a temporary period. At the same time, the organism releases 5-hydroxytryptamine to control pain due to the presence of the pain signal, while also activating GRPR neurons via 5-HT 1A receptors [10] to make the itch sensation more intense. The intense itch sensation elicits a new round of scratching and pain, which then goes into a cycle cycling week after week [59, 75] as shown in Fig. 2.

Second, in 2019 deeper studies on the “itch-scratch vicious cycle” focused on the level above the spinal cord, including the ventral tegmental area (VTA) of the midbrain [6] and the periaqueductal gray (PAG) [76]. Sun Yangang’s group used histamine or chloroquine to provoke the sensation of itching in mice. At the same time, a group of neurons around the periaqueductal gray of mice was traced using neuronal activity labeled molecules, and the neural activity in this brain region was recorded during scratching in mice, finding the electrical activity in this region to be enhanced significantly. Further studies identified a molecular feature shared by this group of neurons that they produce a neuropeptide called tachykinin 1 (Tac1). Mice scratching behavior was obviously reduced when Tac1 neurons were killed or suppressed. In contrast, direct stimulation of these neurons, even without stimulation by pruritogenic agents such as histamine or chloroquine, caused intense scratching behavior in mice spontaneously through the neurons in the downstream link of the spinal cord neural circuit that transfers itch information [76]. Hu’s team, with the help of experimental technical tools at the neural loop level and transgenic mouse models, have explained deeply the role of different types of neurons in the ventral tegmental area of the midbrain reward in the information processing of different signaling pathways of itch, and the results have deepened the understanding of the mechanisms of the central loop of itch perception. Through optogenetic manipulation and conditioned place preference or conditioned place aversion experiments, two types of neurons in the VTA brain region were confirmed to regulate two different components of itch perception, glutamatergic and gamma-aminobutyric acid (GABA)-ergic neurons that mediate the aversion induced by pruritogenic agents and dopaminergic neurons that mediate the pleasure the following scratching. Finally, VTA neurons were found to play similar roles in animal models of chronic pruritus. The findings provide a new perspective on the neural loop mechanism underlying the "itch-scratch-healing scratch cycle" and provide a theoretical and scientific basis for gaining a deeper understanding of the principles of the higher central of itch perception.

Third, the addictive ‘itch-scratch cycle” exacerbates dermatitis and pruritus while also activating reward mechanisms in the central system. The reward system, including the ventral tegmental area (VTA), the nucleus ambiguous (NAc), and the striatum, is essential for brain processing of both addiction and itch [70]. Recent studies in 2021 identified VTA-NAc GABAergic neurons that promote reward-reinforcing behaviors through cholinergic interneurons (CINs) projecting directly to the ventral nucleus accumbens shell (vNAcSh), deepening the understanding of the neuromodulatory interactions between VTA and NAc and furthering the study of addiction mechanisms, providing a new direction for the study of the neural circuit mechanism of brain regions in the acupuncture treatment of "itch-scratch vicious cycle".

## GRPR is a new target of acupuncture for chronic itch by inhibiting the “Itch-Scratch Vicious Cycle”

The possible mechanisms of acupuncture in the treatment of chronic itch based on the spinal cord and above are still at a blank stage, but recent studies have shown that GRPR plays a key role in the conduction process of spinal cord neural pathways in chronic pruritus, and the modulatory effect of acupuncture on GRPR has been initially confirmed. In this paper, we review the studies of the animal mechanism of acupuncture for chronic pruritus, and take the role of GRPR in the “itch-scratch vicious cycle” of chronic pruritus treated by acupuncture as a breakthrough, to provide a possible protocol for acupuncture in the treatment of chronic pruritus to curb the “itch-scratch vicious cycle”.

### Spinal cord level

Gastrin-releasing peptide (GRP) in the dorsal root ganglion of the spinal cord serves as an intermediate link between the peripheral and central “itch-scratch cycle” [77]. GRPR plays an important role in the regulation of itch sensation in the dorsal spinal cord [12, 78]. GRPR is specifically expressed in a small subset of peptidergic dorsal root ganglion neurons, whereas expression of its receptor GRPR is restricted to the dorsal layer I of the spinal cord. Knockout the GRPR gene of mice, itch symptoms are reduced in either histamine-dependent (histamine, 5-HT, compound 48/80) pruritus or non-dependent (protease-activated receptor 2, chloroquine) pruritus. In the future, basic acupuncture research design will be characterized by new technologies, new targets, and multidisciplinary cross-fertilization [79]. Mechanistic studies of acupuncture for chronic pruritus were first performed to validate acupuncture modulation of GRPR, and further adding an intrathecal injection of bombesin-sap to ablate GRPR reverse validation of the role of GRPR neurons in regulating the "itch-scratch vicious cycle" of chronic pruritus. Regarding pruritus model construction, histamine-dependent and non-histamine-dependent pruritus models can be constructed separately using different pruritogenic agents to meet the integrity of the experimental design and to explore the mechanism of acupuncture effect in multi-targets and multi-pathways.

### Above the spinal cord

In the reward system above the spinal cord, the “itch-scratch cycle” focuses on the PAG and VTA brain regions. PAG region as an evolutionarily conserved structure is only activated by both itch and cold pain stimulation [80, 81]. In a human brain imaging study, PAG of the midbrain was activated during noxious cold stimulation to suppress histamine-induced itch [81]. Results of the studies on histamine-induced itch showed significantly stronger neural coupling between the right postcentral insula and the PAG during pruritus [82]. It was shown that initiative scratching is accompanied by higher pleasure and leads to more pronounced deactivation of the anterior cingulate cortex and insula, the process including significant involvement of the reward system such as the midbrain VTA, together with the mechanism of PAG deactivation in the midbrain, suggesting the modulatory effect of pruritus contrary to the mechanisms known to inhibit pain [80]. And one study identified a new population of inhibitory neurons called LJA5 in the mice brainstem that can receive inputs from sensory and stress areas including PAG [83]. It is not difficult to conclude that PAG is activated during itch and is involved in the “itch-scratch cycle” based on the above findings. Sun Yangang’s group found that glutamatergic neurons expressing Tac1 in the lateral and ventral lateral midbrain periaqueductal gray matter (l/vlPAG) facilitated the “itch-scratch cycle”, removing GRPR spinal neurons could inhibit scratching behavior caused by activation of Tac1-expressing neurons [76]. Whether the modulatory effect of acupuncture on GRPR can inhibit the activity of Tac1-expressing neurons to regulate the state of higher central PAG brain regions, improving the “itch-scratch cycle” and exerting antipruritic effects is a new target for future acupuncture treatment of chronic itch.

**Fig. 3:**
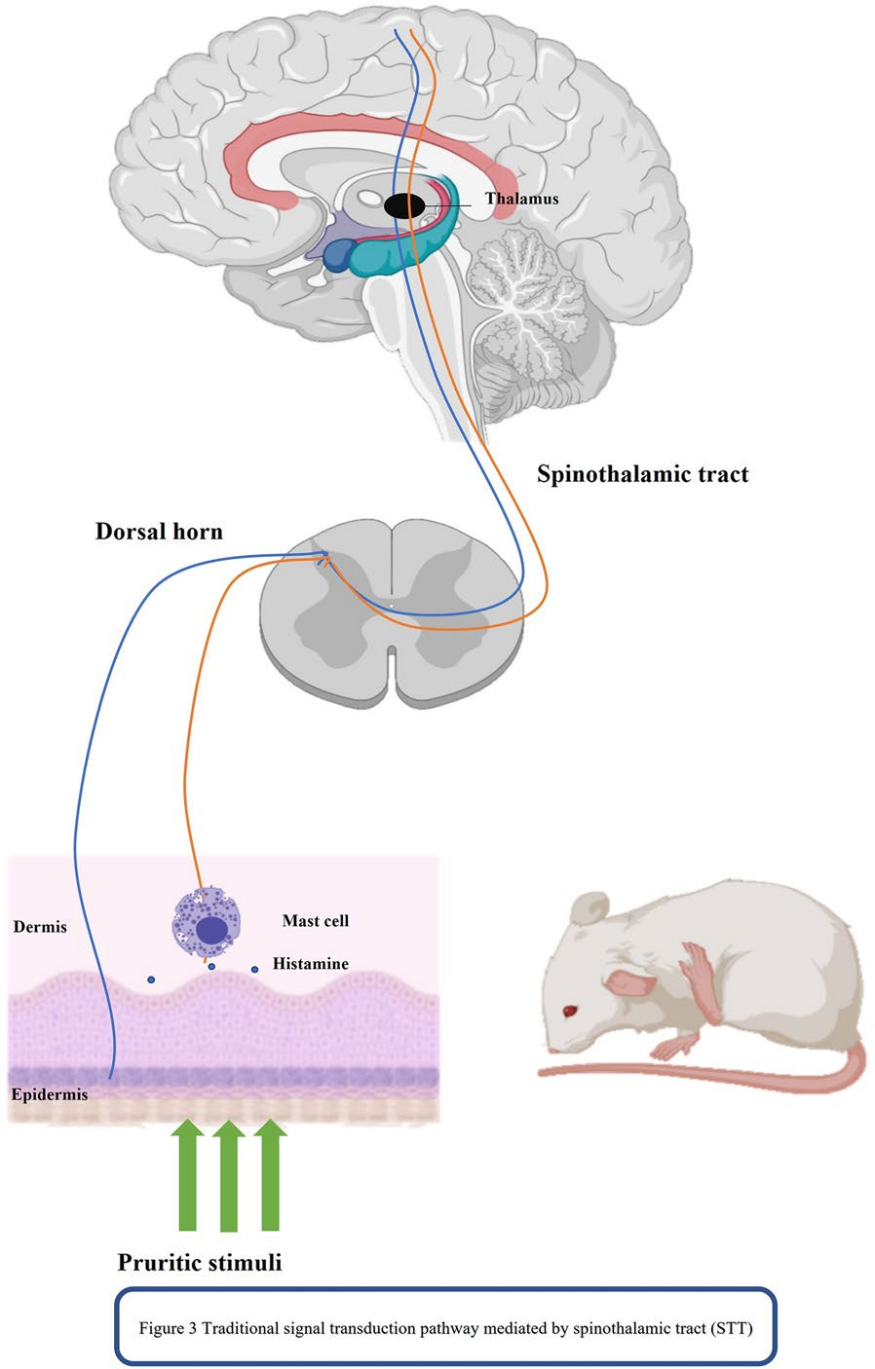
Previous studies of itch pathway have focused on the spinal cord level. Peripheral pruritic mediators stimulate mast cells degranulation in the skin releasing histamine, and histamine-sensitive unmyelinated nerve endings conduct nerve impulses to the posterior horn of the spinal cord, through the spinal thalamic tract to the thalamus, feed backing to the somatosensory cortex pruritus

**Fig. 4:**
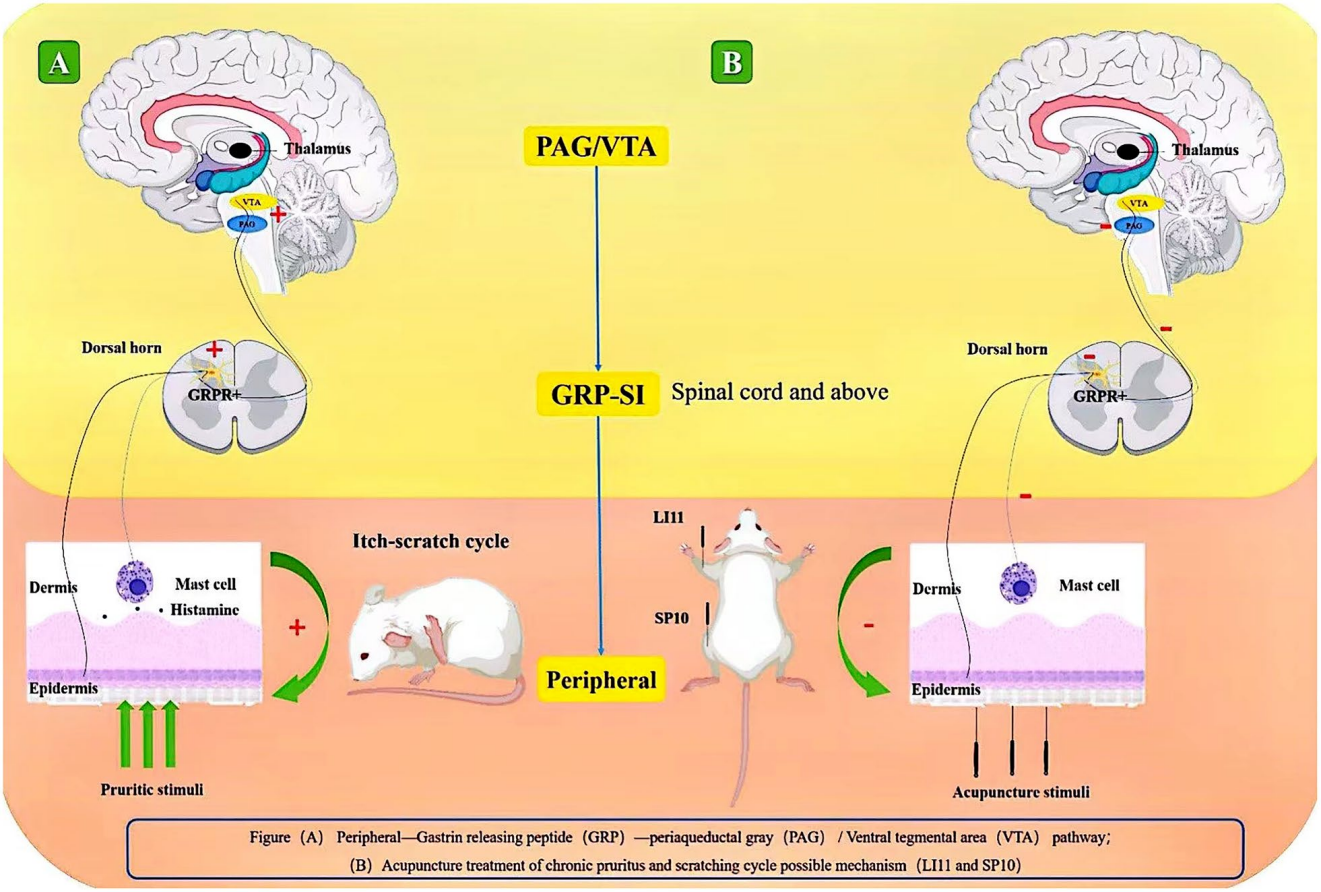
**A** Peripheral-Gastrin releasing peptide-Periaqueductal gray/Ventral tegmental area: Peripheral pruritic mediators activate GRPR + neurons in the superficial layer of the spinal dorsal horn by releasing GRP, nerve impulses uploaded to the VTA and PAG brain regions to feedback itching to the body cortex. **B** Acupuncture treatment of chronic pruritus and scratching cycle possible mechanism: Acupuncture treatment reducing the expression of GRPR in the spinal cord, desensitizing the itch-signaling pathways to VTA and PAG brain regions to relieve chronic itch

During the study of reward mechanisms, the VTA remains the focus of research on the “itch-scratch cycle” and the different neurons of the VTA include dopamine (DA) and GABA neurons [6]. The projections of DA neurons to the striatum, and the striatal circuitry connecting the striatum to the striatum, constitute key components of the brain's reward and reinforcement circuits so changes in VTA activity are significantly associated with scratch-induced pleasure in humans. According to a recent imaging study, the VTA dopamine neurons involved in itch relief reward are similar to those involved in scratch-induced pleasurable behavior in the human midbrain [84]. Glutamatergic and GABAergic neurons mediate aversion induced by itch-causing agents [6], and recent studies have found that GRPR neurons receive local spontaneous excitatory inputs delivered by glutamate and inhibitory inputs from GABA and that all GRPR neurons receive tonic currents induced by glycine and GABA [85]. Therefore, whether GRPR + can play a regulatory role in acupuncture inhibition of the “itch-scratch cycle” by interfering with GABAergic neurons in the VTA deserves further investigation in the future. Previous studies on the “itch-scratch cycle” were mainly based on the thalamic tract of the spinal cord, and the pathway of the occurrence mechanism is shown in Fig. 3. In Fig. 4, the new GRPR marker line and related brain regions demonstrate how acupuncture might interfere with the scratching cycle.

## Summary and future directions

Acupuncture is a safe and effective treatment for pruritus. Increasing understanding of pruritus calls for further investigation into acupuncture’s antipruritic mechanism. Based on the analysis of the studies on acupuncture antipruritics, this paper concluded that GRPR+ is the main target of acupuncture for itch relief. It will provide a good research direction for antipruritic mechanism for acupuncture.

Firstly, concerning the spinal cord and above, GRPR+ may play a significant role in acupuncture regulating the “itch-scratch vicious cycle” to alleviate pruritus symptoms. Secondly, as the PAG- and VTA-based reward centers in the “itch-scratch vicious cycle” of chronic pruritus being studied in greater depth, it will be a major target for future acupuncture treatment of chronic pruritus. Thirdly, the GRPR is an intermediary link in the regulation of the “itch-scratch vicious cycle” by higher centers such as PAG and VTA, to provide a new research pathway for possible mechanisms of acupuncture.

## Supplementary Information

**Additional file 1. **Search strategy.

## Data Availability

Not applicable.

## Abbreviations

GRPR: Gastrin-releasing peptide receptor
VTA: Ventral tegmental area
PAG: Periaqueductal gray matter
KORs: κ-Opioid receptors
GPCR: G protein-coupled receptor
5-HT: 5-Hydroxytryptamine
CSU: Chronic spontaneous urticaria
rs-FC: Resting state functional connectivity
pMCC: Connectivity of the putamen-the posterior part of the midcingulate cortex
SD: Sprague–Dawley
OVA: Ovalbumin
Al (OH)3): Aluminum hydroxide
LI11: Quchi
SP10: Xuehai
LI4: Hegu
ST36: Zusanli
SJ10: Tianjing
SP6: Sanyinjiao
PC6: Neiguan
SJ11: Qinglingyuan
GV20: Baihui
GB20: Fengchi
BL40: Weizhong
BL-17: Geshu
BL13: Feishu
BL67: Zhiyin
DNCB: 2,4-Dinitrochlorobenzene
NK1: Neurokinin-1
IL: Interleukin
TRPV1: Transient receptor potential vanilloid 1
SP: Substance P
IFN-γ: Interferon-gamma
PGE_2_: Prostaglandin E2
Iba1: Ionized calcium-binding adapter molecule 1
β-EP: Beta-endorphinergic
CB: Cannabinoid receptors type
TNF-α: Tumor necrosis factor-α
DYN: Dynorphin
MOR: Mu-opioid receptor
GRP: Gastrin-releasing peptide
BNP: B-type natriuretic peptide
PFC: Prefrontal cortex
SMA: Supplementary motor area
PM: Premotor cortex
vlPAG: Ventrolateral periaqueductal gray
ACC-DMS: Anterior cingulate cortex-dorsomedial striatum
PMC: Primary motor cortex
MC: Middle cingulate cortex
CN: Caudate nucleus
dlPFC: Dorsolateral prefrontal cortex
PCC: Posterior cingulate cortex
Tac1: Tachykinin 1
GABA: Gamma-aminobutyric acid
NAc: Nucleus ambiguous
CINs: Cholinergic interneurons
vNAcSh: Ventral nucleus accumbens shell
l/vlPAG: Lateral and ventral lateral midbrain periaqueductal gray matter
DA: Dopamine
