# Supplementary material for: Role of GRPR in Acupuncture Intervention in the “Itch-scratch Vicious Cycle” Spinal Circuit of Chronic Pruritus
Source: Chin Med. 2023 Jan 3;18:2. doi: 10.1186/s13020-022-00706-4 (PMC9809006; doi:10.1186/s13020-022-00706-4)
Supplement: Supplementary file 1 — Additional file 1. Search strategy. [file 13020_2022_706_MOESM1_ESM.docx]

**Supplementary 1 Search strategy**

**Medline (via Ovid)**

1. exp Acupuncture Therapy/ or exp Acupuncture Points/ or exp Acupuncture/
2. exp Electroacupuncture/
3. exp Meridians/
4. Acupuncture.tw.
5. (electroacupuncture or electro acupuncture).tw.
6. meridian$.tw.
7. needling.tw.
8. acupoint$.tw.
9. or/1-8
10. Pruritus/
11. pruritus.tw.
12. pruritis.tw.
13. pruritic.tw.
14. itch$.tw.
15. scratch.tw.
16. or/10-15
17. 9 and 16

**Embase (via Ovid)**

1. exp Acupuncture Therapy/ or exp Acupuncture Points/ or exp Acupuncture/
2. exp Electroacupuncture/
3. exp Meridians/
4. Acupuncture.tw.
5. (electroacupuncture or electro acupuncture).tw.
6. meridian$.tw.
7. needling.tw.
8. acupoint$.tw.
9. or/1-8
10. Pruritus/
11. pruritus.tw.
12. pruritis.tw.
13. pruritic.tw.
14. itch$.tw.
15. scratch.tw.
16. or/10-15
17. 9 and 16

**Web of science**

1. ((((((((((TS=(exp Acupuncture Therapy/)) OR TS=(exp Acupuncture Points/)) OR TS=(exp Acupuncture/)) OR TS=(exp Electroacupuncture/)) OR TS=(exp Meridians/)) OR TS=(Acupuncture)) OR TS=(electroacupuncture)) OR TS=(electro acupuncture)) OR TS=(meridian$)) OR TS=(needling)) OR TS=(acupoint$)

2. (((((TS=(Pruritus/)) OR TS=(pruritus)) OR TS=(pruritis)) OR TS=(pruritic)) OR TS=(itch$)) OR TS=(scratch)

3. #2 AND #1

**China National Knowledge Infrastructure (CNKI)**

(TKA = 针 OR TKA = 针刺 OR TKA = 电针 OR TKA = 手针 OR TKA = 穴位 OR TKA = 经络) AND (TKA = 瘙痒 OR TKA = 痒 OR TKA = 搔抓)

**Wan Fang Database**

题名或关键词:(针 OR 针刺 OR 电针 OR 手针 OR 穴位 OR 经络) AND 题名或关键词:(瘙痒 OR 痒 OR搔抓)

**VIP Database**

(M=(针 OR 针刺 OR 电针 OR 手针 OR 穴位 OR 经络) OR R=针 OR 针刺 OR 电针 OR 手针 OR 穴位 OR 经络) AND (M=(瘙痒 OR 痒 OR搔抓) OR R=瘙痒 OR 痒 OR搔抓)

**Chinese Biomedical Literature Database (CBM)**

( "针"[常用字段] OR "针刺"[常用字段] OR "电针"[常用字段] OR "手针"[常用字段] OR "穴位"[常用字段] OR "经络"[常用字段]) AND( "瘙痒"[常用字段] OR "痒"[常用字段] OR "搔抓"[常用字段])
